# Factors associated with exclusive breastfeeding at discharge during the COVID-19 pandemic in 17 WHO European Region countries

**DOI:** 10.1186/s13006-022-00517-1

**Published:** 2022-12-02

**Authors:** Ilana Azulay Chertok, Rada Artzi-Medvedik, Maryse Arendt, Emma Sacks, Marina Ruxandra Otelea, Carina Rodrigues, Raquel Costa, Karolina Linden, Mehreen Zaigham, Helen Elden, Daniela Drandic, Susanne Grylka-Baeschlin, Céline Miani, Emanuelle Pessa Valente, Benedetta Covi, Marzia Lazzerini, Ilaria Mariani

**Affiliations:** 1 Ohio University, Athens, OH USA; 2 Ruppin College, Emek Hefer, Israel; 3 Ben-Gurion University of the Negev, Beersheva, Israel; 4 BLL Beruffsverband vun den Laktatiounsberoderinnen zu Lëtzebuerg, Luxembourg, Luxembourg; 5 Johns Hopkins University, Baltimore, MD USA; 6 Carol Davila University of Medicine and Pharmacy, Bucharest, Romania; 7 Instituto de Saude Publica, Universidade Do Porto, Porto, Portugal; 8 Institute of Health and Care Sciences, Sahlgrenska Academy, University of Gothenburg, Gothenburg, Sweden; 9 Skane University Hospital, Lund University, Lund, Sweden; 10 Reproductive Rights Advocacy Program (RODA)- Parents in Action, Zagreb, Croatia; 11 Zurich University of Applied Sciences, Winterthur, Switzerland; 12 School of Public Health, Bielefeld University, Bielefeld, Germany; 13 Institute for Maternal and Child Health, IRCCS “Burlo Garofolo”, Trieste, Italy

**Keywords:** Exclusive breastfeeding, COVID-19 pandemic, International breastfeeding research

## Abstract

**Background:**

Exclusive breastfeeding is the optimal infant nutrition, providing infants immunoprotection against many diseases including SARS-CoV-2 infection. Restrictions during the COVID-19 pandemic may have negatively affected breastfeeding practices in maternity care facilities. The aims of the study were to examine exclusive breastfeeding rates at discharge over time and to identify factors associated with exclusive breastfeeding during the pandemic.

**Methods:**

A cross-sectional survey was conducted among mothers who gave birth in a maternity care facility in the World Health Organization (WHO) European Region countries during the COVID-19 pandemic. The socio-ecological model was employed to examine intrapersonal, interpersonal, organizational, and community/society factors associated with maternal report of exclusive breastfeeding at the time of discharge.

**Results:**

There were 26,709 participating mothers from 17 European Region countries who were included in the analysis. Among the mothers, 72.4% (*n* = 19,350) exclusively breastfed and 27.6% (*n* = 7,359) did not exclusively breastfeed at discharge. There was an overall decline in exclusive breastfeeding rates over time (*p* = 0.015) with a significantly lower rate following the publication of the WHO breastfeeding guidelines on 23 June 2020 (AOR 0.88; 95% CI 0.82, 0.94). Factors significantly associated with exclusive breastfeeding outcomes in the logistic regression analysis included maternal age, parity, education, health insurance, mode of birth, inadequate breastfeeding support, lack of early breastfeeding initiation, lack of full rooming-in, birth attendant, perceived healthcare professionalism and attention, facility room cleanliness, timing of birth, and location of birth.

**Conclusions:**

Results from the study indicate the decline in exclusive breastfeeding rates in the WHO European Region during the COVID-19 pandemic. Using the socio-ecological model to identify factors associated with breastfeeding outcomes facilitates an integrated and holistic approach to address breastfeeding needs among women across the region. These findings demonstrate the need to augment breastfeeding support and to protect exclusive breastfeeding among mother-infant dyads, in an effort to reverse the declining exclusive breastfeeding rates. The study highlights the need to educate mothers and their families about the importance of exclusive breastfeeding, reduce maternal-infant separation, increase professional breastfeeding support, and follow evidence-based practice guidelines to promote breastfeeding in a comprehensive and multi-level manner.

**Trial registration number:**

Clinical Trials NCT04847336.

**Supplementary Information:**

The online version contains supplementary material available at 10.1186/s13006-022-00517-1.

## Background

Exclusive human milk is the physiologically ideal nutrition for infants in the first six months of life, as human milk affords health benefits including reduced risk of infant acute infections such as otitis media, respiratory infections, and gastrointestinal infections [1] as well as support for growth and development [2]. Introduction of artificial alternatives to human milk can disrupt the infant’s microbiota which may increase the risk for poor health outcomes [2]. Moreover, breastfeeding is associated with improved maternal-infant bonding [3]. Yet, despite the extensive evidence in support of exclusive breastfeeding, there are significant differences in exclusive breastfeeding rates in the first 48 h postpartum among the various European countries, ranging from 57.6% in Switzerland to 88.4% in Latvia [4].

The onset of the COVID-19 pandemic in early 2020 posed an urgent need to implement infection prevention measures in maternity care facilities. Particularly in the initial phase of the pandemic, when there was limited evidence-based information on the influence of COVID-19 on mothers and their infants [5], many health systems instituted policies requiring or encouraging isolation of infants and limitations on family inclusion and visitation. Reports indicate that many facilities did not consistently prioritize skin-to-skin care and breastfeeding [6], despite being recommended by evidence-based professional guidelines [7] and the World Health Organization (WHO) [8]. Early in the pandemic, there were rapidly changing guidelines and conflicting recommendations on care for infants born to mothers who were suspected of or tested positive for SARS-CoV-2, leading to unnecessary mother-infant separation [9]. Separation of mothers and infants, restrictions on birth companions, and bans on visitors were common in many facilities throughout the WHO European Region [10]. Maternal-infant restrictions were contrary to many international regulations, challenging the rights of mothers and infants [11].

The WHO and the United National Children’s Fund (UNICEF) [12] have promoted the revised Baby Friendly Hospital Initiative (BFHI) as a global program since its original version in 1991, to encourage maternity care facilities worldwide to implement the Ten Steps for Successful Breastfeeding. The recommendations include maternal-infant care supportive of breastfeeding such as skin-to-skin contact, early initiation and exclusive breastfeeding, and rooming-in. Implementation of the BFHI Ten Steps has positively influenced early breastfeeding initiation, exclusive breastfeeding at facility discharge, and duration of exclusive and any breastfeeding, with a dose–response association [13]. Early skin-to-skin contact has been associated with significantly higher rates of exclusive breastfeeding [14].

On 13 March 2020, the WHO published interim guidelines regarding clinical management of mothers and infants with COVID-19 that stated the importance of initiating breastfeeding even if the mother had suspected, probable, or confirmed COVID-19 [15], and updated the guidelines on 23 June 2020 [8]. Recent research has demonstrated that human milk contains IgA and IgG antibodies against COVID-19 thereby affording specific immunoprotection against the virus and neutralized viral activity [16], suggesting not only low risk of COVID-19 transmission through human milk but potential protection in affected dyads. Evidence suggests that restrictions imposed to mitigate the spread of COVID-19 such as maternal-infant separation and lack of skin-to-skin contact may have negatively affected breastfeeding practices during the pandemic [17, 18].

Exclusive breastfeeding at discharge is one of the measures of the quality of facility-based maternity care [19]. The primary aim of the current study was to examine exclusive breastfeeding rates at discharge over time, with specific focus on changes following the publication of the WHO COVID-19 breastfeeding guidelines on 23 June 2020. The secondary aim was to identify factors significantly associated with exclusive breastfeeding at facility discharge during the COVID-19 pandemic in the European Region.

## Methods

The current study of 17 participating countries is part of IMAgiNE EURO [20], a larger cross-sectional survey study conducted in 20 countries of the WHO European Region according to the General Data Protection Regulation (GDPR). The study design followed the Strengthening the Reporting of Observational Studies in Epidemiology (STROBE) guidelines for cross-sectional studies [21]. The study protocol was approved by the institutional review board of the coordinating center and then reviewed and approved or deemed exempt by the ethics committees of other participating researchers’ countries.

Prior to participation in the online survey, consent was obtained after mothers were informed of the study objectives, methods, and their right to decline participation (the data protection policy was available for download). Anonymity was ensured by not collecting identifying information. Data transmission and storage were secured by encryption.

Collaborating researchers distributed a link to an anonymous and voluntary online survey. The survey was made available in multiple languages with the option to participate using the participant’s preferred language regardless of geographic location. Participating countries used different dissemination strategies for recruitment of participants including social media, organizational websites, and local networks. Detailed description of the survey and its development has been previously reported [20].

The survey was based on the domains of the WHO Standards for Improving Quality of Maternal and Newborn Care in Health Facilities [22], namely the provision of care, experience of care, and availability of motivated human resources and essential physical resources, with an additional domain on key organizational changes related to the COVID-19 pandemic and questions on sociodemographic characteristics. Breastfeeding practices were included as part of the provision of care domain and were assessed through items referring to the organizational context, support, and exclusive breastfeeding outcomes.

### Data analysis

Data were cleaned according to a previously agreed protocol [20]. Mothers met the inclusion criteria if they were at least 18 years old, gave birth to a live, singleton infant in a facility in the WHO European Region countries between 1 March 2020 and 28 February 2022, consented to participate in the survey, and answered all 40 quality measures including the main outcome of maternal report of breastfeeding at the time of discharge (with no provision of formula) and five key indicators of date of birth, age, education, parity, and whether the women gave birth in the same country where she was born. Possible duplicates were detected and excluded. Countries with a minimum of 300 participants were included in the analysis, based on the primary outcome of the estimated exclusive breastfeeding rate after birth of 75% ± 5% [23] and 5% type I error. For purposes of this study, home births, stillbirths, twin or multiple births, and infants admitted to the neonatal intensive care unit (NICU) or special care baby unit (SCBU) were excluded from the analysis (thereby excluding preterm and severely ill infants).

The primary outcome of interest was exclusive breastfeeding at discharge, analyzed as a dichotomous variable and defined as exclusive breastfeeding versus partial or no breastfeeding (non-exclusive breastfeeding). We examined differences in exclusive breastfeeding rates at discharge over time during the pandemic overall and for each country using the Cochran-Armitage trend test. Rates were compared before and after the publication of the WHO COVID-19 breastfeeding guidelines [8] on 23 June 2020 to examine differences based on recommendations. For each country, we conducted a trend analysis comparing 4-month time periods (Period 1: 1 March 2020 to 30 June 2020; Period 2: 1 July 2020 to 30 October 2020; Period 3: 1 November 2021 to 28 February 2021; Period 4: 1 March 2021 to 30 June 2021; Period 5: 1 July 2021 to 30 October 2021; Period 6: 1 November 2021 to 28 February 2022).

For the secondary study aim, relevant variables to exclusive breastfeeding were included in the analysis according to the socio-ecological model theoretical framework which has been previously used in breastfeeding studies [24, 25]. The independent variables were grouped according to the model’s domains. Intrapersonal factors were socio-demographic factors of maternal age, parity, education, payment of maternity care, giving birth in same country as mother’s origin and prenatal/birth factors during the COVID-19 pandemic including difficulty attending routine prenatal care, faced barriers to prenatal care (including logistic, financial, lockdown, and lack of childcare), mode of birth, and maternal ICU admission. Interpersonal factors related to social support of the mother are represented by adequate visiting hours for partner or relatives, presence of companion of choice, perceived emotional support from healthcare provider, adequate breastfeeding support, effective communication, maternal involvement in healthcare decisions, treatment with dignity, and not experiencing abuse. Organizational factors included skin-to-skin contact within the first hour, early breastfeeding initiation within the first hour, rooming-in, infant allowed to stay with mother as wished, type of healthcare provider at birth, perceived adequate number of healthcare providers given the workload, adequate assistance from healthcare providers, professionalism of healthcare providers, immediate attention by healthcare providers when needed, privacy protected by healthcare providers, type of maternity care facility (public or private), adequate number of mothers per room, comfort of facility room, and room cleaning. For the community and society factors variables included were timing of birth (from publication of the WHO COVID-19 breastfeeding guidelines on 23 June 2020), country of infant birth, and country’s proportion of facilities ever BFHI-accredited [26].

Descriptive analysis was conducted to calculate frequencies and proportions. Chi-square tests were used to assess differences in exclusive breastfeeding for each variable and to determine the variables to include in the regression analysis. Multivariable logistic regression was employed to identify the variables significantly associated with exclusive breastfeeding, using an iterative stepwise variable selection. Adjusted odds ratio (AOR) and 95% confidence interval (CI) were reported for each independent variable. Additionally, for mothers who reported their COVID-19 status, a sub-group analysis using chi-square tests was conducted to compare differences in exclusive breastfeeding rates among mothers infected or suspected of infection to determine the influence of the COVID-19 on early postpartum breastfeeding practices. All tests were two-sided and p < 0.05 was considered statistically significant. Statistical analyses were conducted in Stata version 14 and R version 4.1.1.

## Results

**Fig. 1:**
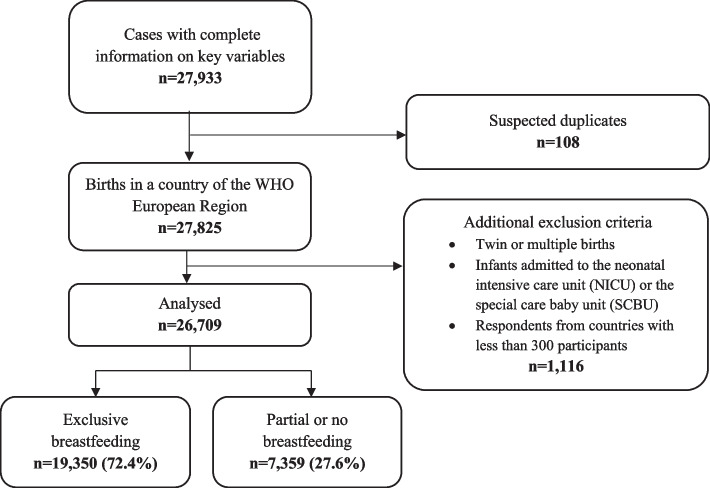
Flow diagram of sample included in analysis, based on maternal reported exclusive breastfeeding and partial or no breastfeeding status at discharge

A sample of 26,709 mothers who gave birth in a facility to a healthy, singleton infant during the COVID-19 pandemic in 17 countries of the WHO European Region was included in the analysis using 45 key variables, 40 key quality measures including the main outcome of infant feeding at discharge, and five key socio-demographic items (see Fig. 1).

**Fig. 2:**
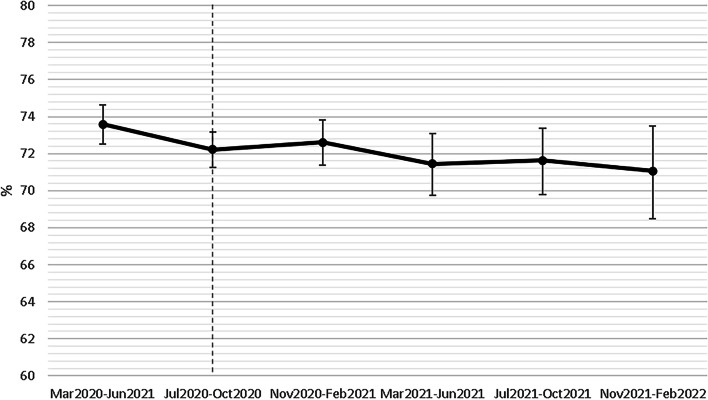
Trend analysis of exclusive breastfeeding over time (*n* = 26,709). (Note: Scale is presented on 60 to 80 range. The vertical line represents the publication of the WHO breastfeeding guidelines in June 2020.)

**Fig. 3:**
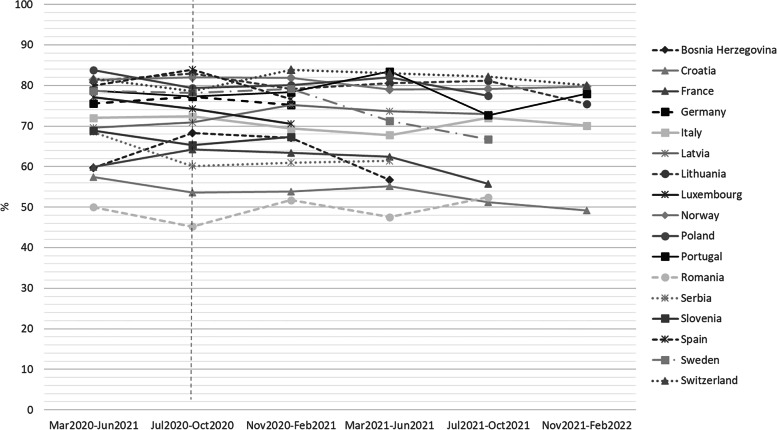
Trend analysis of exclusive breastfeeding for each country over time (*n* = 26,709). (Note: The vertical line represents the publication of the WHO breastfeeding guidelines in June 2020. Data for the last four periods were not shown for all countries because of low sample (*N* < 40).)

Among the mothers, 72.4% exclusively breastfed, 23.0% partially breastfed, and 4.6% gave formula only at discharge (for a combined total percent of 27.6% not being exclusively breastfed at discharge). There was a significantly decreasing trend in overall exclusive breastfeeding rates over time (*p* = 0.015) (Fig. 2) with non-significant discrepancies between countries (Fig. 3).

Based on the socio-ecological model, descriptive statistics of the intrapersonal, interpersonal, organizational, and community and society factors are presented according to breastfeeding practices (Table 1) with presentation of additional information on intrapersonal factors by country (Supplementary Tables 1 and 2).

**Table 1:** Characteristics of the overall sample of mothers who gave birth during the COVID-19 pandemic in the WHO European Region, by factors included in the socio-ecological model (*n* = 26,709)

	**Exclusive breastfeeding** ***N* = 19,350 (72.4%)**	**Partial or no breastfeeding** ***N* = 7,359 (27.6%)**	***P*-value**
**Intrapersonal factors**			
***Sociodemographic factors***			
*Maternal age*			
18–24	910 (4.7)	456 (6.2)	< 0.001
25–30	6839 (35.3)	2586 (35.1)	0.756
31–35	7914 (40.9)	2807 (38.1)	< 0.001
36–39	2901 (15.0)	1130 (15.4)	0.459
40 or older	786 (4.1)	380 (5.2)	< 0.001
*Parity*			
Primipara	10,575 (54.7)	5189 (70.5)	< 0.001
Multipara	8775 (45.3)	2170 (29.5)	
*Maternal education*			
Junior high school or lower	945 (4.9)	518 (7.0)	< 0.001
High school	4313 (22.3)	1916 (26.0)	< 0.001
University degree	7527 (38.9)	2735 (37.2)	0.009
Graduate degree (Master/Doctorate)	6565 (33.9)	2190 (29.8)	< 0.001
*Payment of maternity care (insurance status)*			
Non-private insurance (public/state/employer)	18,634 (96.3)	6913 (93.9)	< 0.001
Private insurance	467 (2.4)	208 (2.8)	0.055
No insurance (self-paid)	249 (1.3)	238 (3.2)	< 0.001
*Infant born in same country as mother’s origin*			
Yes	17,989 (93.0)	6837 (92.9)	0.886
No	1361 (7.0)	522 (7.1)	
***Prenatal and birth factors***			
*Difficulty attending prenatal care*			
Yes, always/Nearly always	1380 (7.1)	585 (7.9)	0.022
Sometimes	5765 (29.8)	2322 (31.6)	0.005
No, never/Almost never	12,205 (63.1)	4452 (60.5)	< 0.001
*Faced barriers to access prenatal care*			
Yes, always/Nearly always	1010 (5.2)	438 (6.0)	0.018
Sometimes	4586 (23.7)	1777 (24.1)	0.444
No, never/Almost never	13,754 (71.1)	5144 (69.9)	0.058
*Mode of birth*			
Spontaneous vaginal birth	14,762 (76.3)	4547 (61.8)	< 0.001
Instrumental vaginal birth	1522 (7.9)	592 (8.0)	0.628
Cesarean birth	3066 (15.8)	2220 (30.2)	< 0.001
*Maternal ICU admission*			
Yes	92 (0.5)	84 (1.1)	< 0.001
**Interpersonal factors**			
***Social support***			
*Adequate visiting hours for partner/relatives*			
Excellent/good	4798 (24.8)	1418 (19.3)	< 0.001
Sufficient	3089 (16.0)	956 (13.0)	< 0.001
Insufficient/very bad	11,463 (59.2)	4985 (67.7)	< 0.001
*Presence of companion of choice*			
Yes, always/Nearly always	7879 (40.7)	2436 (33.1)	< 0.001
Sometimes	3309 (17.1)	1041 (14.1)	< 0.001
No, never/Almost never	8162 (42.2)	3882 (52.8)	< 0.001
*HCP emotionally supportive*			
Yes, always/Nearly always	14,155 (73.2)	4652 (63.2)	< 0.001
Sometimes	3523 (18.2)	1710 (23.2)	< 0.001
No, never/Almost never	1672 (8.6)	997 (13.5)	< 0.001
*Effective communication from HCP*			
Yes, always/Nearly always	14,037 (72.5)	4334 (58.9)	< 0.001
Sometimes	4489 (23.2)	2376 (32.3)	< 0.001
No, never/Almost never	824 (4.3)	649 (8.8)	< 0.001
*Maternal involvement in healthcare decisions*			
Yes, always/Nearly always	12,779 (66.0)	3914 (53.2)	< 0.001
Sometimes	4595 (23.7)	2129 (28.9)	< 0.001
No, never/Almost never	1976 (10.2)	1316 (17.9)	< 0.001
*Treated with dignity*			
Yes, always/Nearly always	15,157 (78.3)	4828 (65.6)	< 0.001
Sometimes	3528 (18.2)	2035 (27.7)	< 0.001
No, never/Almost never	665 (3.4)	496 (6.7)	< 0.001
*Abuse* (physical /verbal/emotional)			
Yes, always/Nearly always	434 (2.2)	240 (3.3)	< 0.001
Sometimes	2011 (10.4)	1185 (16.1)	< 0.001
No, never/Almost never	16,905 (87.4)	5934 (80.6)	< 0.001
**Organizational factors**			
*Skin-to-skin contact in the first hour after birth*			
Yes	17,173 (88.7)	5657 (76.9)	< 0.001
*Early breastfeeding*			
Yes	2612 (13.5)	2465 (33.5)	< 0.001
*Adequate breastfeeding support*			
Yes	5196 (26.9)	3353 (45.6)	< 0.001
*Rooming-in*			
Full rooming-in (including night)	16,814 (86.9)	5507 (74.8)	< 0.001
Mostly rooming-in	1752 (9.1)	1321 (17.9)	< 0.001
Mostly/always in healthy infant nursery	784 (4.0)	531 (7.2)	< 0.001
*Allowed to stay with infant as long as wished*			
Yes	861 (4.4)	600 (8.2)	< 0.001
*HCP type present at birth*			
Midwife	17,481 (90.3)	6172 (83.9)	< 0.001
Nurse	7067 (36.5)	2920 (39.7)	< 0.001
Student (before graduation)	2835 (14.7)	923 (12.5)	< 0.001
Obstetrics post-graduate registrar/resident	3149 (16.3)	1454 (19.8)	< 0.001
Obstetrics physician	10,150 (52.5)	4557 (61.9)	< 0.001
I don’t know	1774 (9.2)	943 (12.8)	< 0.001
Other	2277 (11.8)	758 (10.3)	0.001
*Adequate number of HCP given the workload*			
Excellent/good	8884 (45.9)	2724 (37.0)	< 0.001
Sufficient	6915 (35.7)	2765 (37.6)	0.005
Insufficient/very bad	3551 (18.4)	1870 (25.4)	< 0.001
*Adequate assistance from HCP*			
Yes, always/Nearly always	13,864 (71.6)	4520 (61.4)	< 0.001
Sometimes	4274 (22.1)	2063 (28.0)	< 0.001
No, never/Almost never	1212 (6.3)	776 (10.5)	< 0.001
*HCP professionalism*			
Excellent/good	12,931 (66.8)	3818 (51.9)	< 0.001
Sufficient	5542 (28.6)	2850 (38.7)	< 0.001
Insufficient/very bad	877 (4.5)	691 (9.4)	< 0.001
*Immediate attention by HCP when needed*			
Yes, always/Nearly always	14,036 (72.5)	4415 (60.0)	< 0.001
Sometimes	4433 (22.9)	2264 (30.8)	< 0.001
No, never/Almost never	881 (4.6)	680 (9.2)	< 0.001
*Privacy protected by HCP*			
Yes, always/Nearly always	15,374 (79.5)	5364 (72.9)	< 0.001
Sometimes	2707 (14.0)	1270 (17.3)	< 0.001
No, never/Almost never	1269 (6.6)	725 (9.9)	< 0.001
*Type of birth facility*			
Public	17,912 (92.6)	6608 (89.8)	< 0.001
Private	1438 (7.4)	751 (10.2)	
*Adequate number of women per room*			
Excellent/good	13,492 (69.7)	4790 (65.1)	< 0.001
Sufficient	4318 (22.3)	1858 (25.2)	< 0.001
Insufficient/very bad	1540 (8.0)	711 (9.7)	< 0.001
*Comfort of facility room*			
Good/excellent	9988 (51.6)	3139 (42.7)	< 0.001
Sufficient	7857 (40.6)	3318 (45.1)	< 0.001
Insufficient/very bad	1505 (7.8)	902 (12.3)	< 0.001
*Room cleaning*			
Excellent/good	13,034 (67.4)	4434 (60.3)	< 0.001
Sufficient	5277 (27.3)	2224 (30.2)	< 0.001
Insufficient/very bad	1039 (5.4)	701 (9.5)	< 0.001
**Community and society factors**			
*Timing*			
Birth before June 23, 2020	5221 (37.0)	1889 (25.7)	0.031
Birth from June 23, 2020	14,129 (73.0)	5470 (74.3)	
*Country of infant’s birth*			
Bosnia and Herzegovina	227 (1.2)	132 (1.8)	< 0.001
Croatia	836 (4.3)	719 (9.8)	< 0.001
France	649 (3.4)	397 (5.4)	< 0.001
Germany	687 (3.6)	216 (2.9)	0.013
Italy	4109 (21.2)	1662 (22.6)	0.017
Latvia	1186 (6.1)	442 (6.0)	0.707
Lithuania	682 (3.5)	167 (2.3)	< 0.001
Luxemburg	292 (1.5)	106 (1.4)	0.679
Norway	2178 (11.3)	509 (6.9)	< 0.001
Poland	1152 (6.0)	280 (3.8)	< 0.001
Portugal	1372 (7.1)	387 (5.3)	< 0.001
Romania	413 (2.1)	436 (5.9)	< 0.001
Serbia	466 (2.4)	272 (3.7)	< 0.001
Slovenia	1212 (6.3)	604 (8.2)	< 0.001
Spain	231 (1.2)	52 (0.7)	0.001
Sweden	2966 (15.3)	823 (11.2)	< 0.001
Switzerland	692 (3.6)	155 (2.1)	< 0.001
*Country’s percent of facilities ever BFHI designated*			
Not Reported or 0–49%	11,465 (59.3)	4300 (58.4)	0.224
50–100%	7885 (40.7)	3059 (41.6)	

*Note*: Exclusive breastfeeding (with no provision of formula) was based on maternal report at the time of discharge. Chi-square tests were performed to assess differences between the groups. For variables with more than two categories, each single category was tested against all other categories combined

*Abbreviations: BFHI* Baby Friendly Hospital Initiative, *HCP* health care provider

Among the 14,963 (56.0%) respondents reporting COVID-19 infection status, 1,138 (7.6%) had been infected or suspected of infection and 13,825 (92.4%) had not tested positive for the infection during pregnancy, birth, or postpartum facility stay. Exclusive breastfeeding rates did not significantly differ based on COVID-19 status (*p* = 0.101).

Results of the multivariable logistic regression model demonstrate that factors significantly associated with exclusive breastfeeding were maternal age of 25–30 years (AOR 1.12; 95% CI 1.05, 1.20), multiparity (AOR 1.93; 95% CI 1.80, 2.06), graduate education (AOR 1.08; 95% CI 1.01, 1.17), and giving birth in particular countries (Poland, Serbia, and Spain) (Table 2). Factors associated with lack of exclusive breastfeeding included maternal age of 36–39 years and 40 or older (AOR 0.83; 95% CI 0.76, 0.91 and OR 0.71; 95% CI 0.62, 0.82, respectively), private health insurance (AOR 0.75; 95% CI 0.62, 0.91), no health insurance (AOR 0.56; 95% CI 0.46, 0.69), cesarean birth (AOR 0.65; 95% CI 0.60, 0.70), perceived inadequate breastfeeding support (AOR 0.61; 95% CI 0.57, 0.66), lack of early breastfeeding initiation (AOR 0.53; 95% CI 0.49, 0.57), partial rooming-in (AOR 0.66; 95% CI 0.61, 0.72) or no rooming-in (AOR 0.76; 95% CI 0.66, 0.87), obstetrics physician attending birth (AOR 0.85; 95% CI 0.80, 0.91), perceived that healthcare provider professionalism was sufficient or insufficient/very bad (compared to excellent or good) (AOR 0.80; 95% CI 0.75, 0.86 and AOR 0.75; 95% CI 0.66, 0.86, respectively), perceived lack of immediate attention when needed (AOR 0.80; 95% CI 0.70, 0.92), insufficient/bad cleanliness of room (AOR 0.84; 95% CI 0.75, 0.95), timing of birth from 23 June 2020 to 28 February 2022 (AOR 0.88; 95% CI 0.82, 0.94), and giving birth in particular countries (Croatia, France, Latvia, Luxemburg, Romania, and Slovenia) (Table 2).

**Table 2:** Factors associated with exclusive breastfeeding, results of multivariable logistic regression (*n* = 26,709)

	**AOR (95% CI)**	***P*-value**
**Intrapersonal factors**		
***Sociodemographic factors***		
*Age (years)*		
18–24	1.14 (1.00–1.32)	0.055
25–30	1.12 (1.05–1.20)	0.001
31–35	Ref	
36–39	0.83 (0.76–0.91)	< 0.001
40 or older	0.71 (0.62–0.82)	< 0.001
*Parity*		
Primipara	Ref	
Multipara	1.93 (1.80–2.06)	< 0.001
*Maternal education*		
Junior high school or lower	0.60 (0.53–0.68)	< 0.001
High school	0.75 (0.69–0.81)	< 0.001
University degree	Ref	
Graduate degree (Master/Doctorate)	1.08 (1.01–1.17)	0.032
*Payment of maternity care (insurance status)*		
Non-private insurance (public/state/employer)	Ref	
Private insurance	0.75 (0.62–0.91)	0.003
No insurance (self-paid)	0.56 (0.46–0.69)	< 0.001
***Prenatal and birth factors***		
*Mode of birth*		
Spontaneous vaginal birth	Ref	
Instrumental vaginal birth	0.97 (0.87–1.09)	0.612
Cesarean birth	0.65 (0.60–0.70)	< 0.001
**Interpersonal factors**		
*Adequate breastfeeding support*		
Yes	Ref	
No	0.61 (0.57–0.66)	< 0.001
**Organizational factors**		
*Early breastfeeding*		
Yes	Ref	
No	0.53 (0.49–0.57)	< 0.001
*Rooming-in*		
Full rooming-in (including night)	Ref	
Mostly rooming-in	0.66 (0.61–0.72)	< 0.001
Mostly/always in healthy infant nursery	0.76 (0.66–0.87)	< 0.001
*HCP type present at birth: obstetrics physician*		
Yes	0.85 (0.80–0.91)	< 0.001
No	Ref	
*HCP professionalism*		
Excellent/good	Ref	
Sufficient	0.80 (0.75–0.86)	< 0.001
Insufficient/very bad	0.75 (0.66–0.86)	< 0.001
*Immediate attention by HCP when needed*		
Yes, always/Nearly always	Ref	
Sometimes	0.93 (0.86–1.00)	0.059
No, never/Almost never	0.80 (0.70–0.92)	0.001
*Room cleaning*		
Excellent/good	Ref	
Sufficient	0.99 (0.93–1.06)	0.808
Insufficient/very bad	0.84 (0.75–0.95)	0.004
**Community and society factors**		
*Timing: Birth from June 23, 2020*		
No	Ref	
Yes	0.88 (0.82–0.94)	< 0.001
*Country of infant’s birth*		
Bosnia and Herzegovina	0.98 (0.76–1.24)	0.840
Croatia	0.46 (0.41–0.53)	< 0.001
France	0.41 (0.35–0.47)	< 0.001
Germany	0.89 (0.75–1.06)	0.186
Italy	Ref	
Latvia	0.69 (0.6–0.78)	< 0.001
Lithuania	1.13 (0.94–1.37)	0.198
Luxemburg	0.66 (0.52–0.84)	0.001
Norway	1.08 (0.95–1.22)	0.235
Poland	1.39 (1.19–1.64)	< 0.001
Portugal	1.11 (0.97–1.28)	0.141
Romania	0.69 (0.58–0.82)	< 0.001
Serbia	1.22 (1.01–1.47)	0.041
Slovenia	0.56 (0.49–0.63)	< 0.001
Spain	1.41 (1.02–1.95)	0.038
Sweden	0.95 (0.85–1.05)	0.303
Switzerland	1.2 (0.99–1.47)	0.063

*Abbreviations: HCP* health care provider, *AOR* adjusted odds ratio

## Discussion

Exclusive breastfeeding, even and possibly especially during the COVID-19 pandemic, is beneficial for mothers and infants. Yet, evidence from the study indicates declining rates and identifies factors that are barriers to exclusive breastfeeding during the pandemic. According to the socio-ecological model, intrapersonal, interpersonal, organizational, community and society factors were significantly associated with exclusive breastfeeding during the pandemic, as breastfeeding practices are multifactorial. In our study, several intrapersonal factors were associated with exclusive breastfeeding, including maternal age, parity, education, health insurance, and mode of birth. Older mothers were less likely to exclusively breastfeed compared to younger mothers, which is supported by a population-based study in Spain conducted prior to the pandemic [14], but differed from a study Poland where older mothers were more likely to exclusively breastfeed [27]. In our study, multiparous mothers were more likely to exclusively breastfeed at discharge, similar to an online study conducted during the pandemic in the United Kingdom which found that multiparous women were more likely to breastfeed [28]. Health insurance is one of the variables representing socioeconomic status relevant to the individual intrapersonal factors [25], although mechanisms of payment for healthcare services differs among European countries. Consistent with previous research conducted in Italy [29], Croatia [30], and Romania [31], higher maternal education was associated with higher likelihood of exclusive breastfeeding, whereas cesarean birth was associated with non-exclusive breastfeeding at discharge.

The interpersonal factor significantly associated with exclusive breastfeeding was maternal perception of adequate breastfeeding support. In a sub-analysis from one participating country in the larger European study, over 36% of mothers reported inadequate breastfeeding support during the pandemic [32]. The employment of lactation professionals has consistently contributed to improved breastfeeding outcomes [24], but their availability may have been limited during the pandemic. Other social factors were not significantly associated with exclusive breastfeeding in our study, which may relate to the pandemic circumstances that restricted family members from visiting and attending to the mother and her infant, thereby placing more importance on the support from healthcare providers.

The organizational factors of rooming-in and early breastfeeding were significantly associated with exclusive breastfeeding outcomes. Similarly, in a sub-analysis of the original study’s participants in Sweden, over 16% reported not having full rooming-in [32]. Type of healthcare provider at birth, professionalism, and immediacy of attention were associated with exclusive breastfeeding in the multivariable model, suggesting the important role of healthcare providers in breastfeeding practices. This may be explained by the critical role of trained professionals in providing breastfeeding support during the early postpartum period, as found among mothers giving birth in Italy [28] and in Croatia [30]. A multi-country meta-synthesis pointed to the organizational factors positively influencing breastfeeding support and outcomes such as midwifery care and person-centered communication [33]. A qualitative study in Belgium found that midwives perceived their roles as providing mothers with breastfeeding education and support, although they faced barriers in the facility setting [34].

An additional organizational factor was the association between maternal perception of room cleanliness and exclusive breastfeeding, which was a novel finding. Clean lactation space in the workplace has been shown to influence continued breastfeeding [35], suggestive of a similar organizational factor. Furthermore, during the pandemic, there was a heightened need for cleanliness and hygiene in health facilities.

In a previous study that employed the socio-ecological model, breastfeeding supportive policies and practices were identified as organizational level facilitators [24]. Early breastfeeding and full rooming-in are BFHI practices associated with exclusive breastfeeding. Rooming-in is foundational in minimizing maternal-infant separation [36]. Separating mothers and their infants who had tested positive or were suspected of COVID-19, negatively influenced breastfeeding and was associated with maternal distress [18]. Giving birth during the pandemic in BFHI accredited facilities was associated with higher exclusive breastfeeding rates, higher likelihood of skin-to-skin contact, and lower rates of maternal-infant separation [37]. Less than half of the participating countries reported at least 50% of their maternity care facilities had been BFHI designated, similar to findings in another WHO European Region multi-country study [38], suggesting a need for increasing implementation of the global effort to promote breastfeeding.

Community and society factors were represented by timing of breastfeeding guidelines and country of birth. Data collected prior to the pandemic in the WHO European Region highlight differences in early initiation of breastfeeding and exclusive breastfeeding rates among the member countries [38]. In the early weeks of the pandemic, guidelines were continually being revised based on updated findings. On 23 June 2020, the WHO released its second set of guidelines regarding clinical management of infants and mothers with COVID-19 infection which were more protective of breastfeeding than previous guidelines published during the pandemic [8]. A study in Spain found that exclusive breastfeeding rates at discharge among mothers who had COVID-19 infection at birth were higher in BFHI accredited facilities, where the implementation of skin-to-skin and rooming-in practices was higher than in other facilities [37]. Our study supports previous findings showing differences in exclusive breastfeeding rates specific to particular countries, of which there are varying levels of BFHI accredited facilities and adherence to BFHI policies [23, 26].

A concerning finding of the study is the declining trend in exclusive breastfeeding at discharge, despite the WHO recommendations. This phenomenon was observed by other researchers in Europe who found that facilities restricted breastfeeding support early in the pandemic, resulting in inadequacy and inaccessibility of breastfeeding support [39]. Our study adds to the literature by tracking the continued decline in exclusive breastfeeding rates extended over time, even after the updated WHO recommendations. In a survey of 124 European healthcare facilities who reported BFHI practices during the pandemic, 6% recommended formula rather than breastfeeding for mothers infected with COVID-19 [40] which may contribute to the overall decreased exclusive breastfeeding rates in the European Region. Additionally, with the outbreak of the pandemic, many professionals adapted their lactation services to offer virtual support which facilitated remote access although there were challenges with connection, communication, reading body language, accuracy of assessment, and providing assistance [41].

### Limitations and strengths

Limitations of the study include voluntary maternal self-report with possible selection and reporting biases. Additionally, the survey did not inquire into the infant sex, birth weight, and gestational age, as the focus of the original study was on the maternal perception of quality of care. Gestational age is often a significant factor in breastfeeding outcomes [42], but considering the association of rooming-in on early exclusive breastfeeding [43], singleton infants who were not admitted to the NICU or SCBU served as a proxy for “low risk” infants which would exclude preterm or sick infants. The survey question regarding COVID-19 infection or suspected infection did not inquire into the timing of the infection during pregnancy, birth, or early postpartum which precluded an in-depth analysis of the association of timing of COVID-19 status and exclusive breastfeeding outcomes. Finally, the survey did not inquire into the current or previous BFHI accreditation status of facilities, so we accounted for BFHI status through country-level reporting. Despite the limitations, this study provides a multi-country analysis of exclusive breastfeeding at discharge over the first two years of the COVID-19 pandemic in 17 countries of the WHO European Region. The survey was developed according to the WHO Standards and therefore allows for comparison across countries and sub-groups and the large sample size provides confidence in the findings.

## Conclusions

Findings from the current study highlight the utility of the socio-ecological model in identifying facilitators and barriers to exclusive breastfeeding at discharge and in informing the development of a comprehensive, multi-level approach to breastfeeding promotion within country-specific contexts, to support maternal-infant health throughout the WHO European Region during and following the pandemic. Study findings indicate the need to enhance breastfeeding promotion and support, especially considering the declining rates of exclusive breastfeeding during the COVID-19 pandemic. Effort and investment should be made to increase professional support of breastfeeding, in-patient and post-discharge, to enhance maternal-infant health. Furthermore, results of the study suggest the need to augment breastfeeding support and to continue participation in international reporting. Consistent reporting using defined measures, indicators, and methods can facilitate the monitoring and assessment of the quality of breastfeeding services and outcomes among WHO European Region countries with the overall aim of protecting breastfeeding.

## Supplementary Information

**Additional file 1: Supplementary Table 1.** Intrapersonal factors of the mothers who reported exclusive breastfeeding at the time of discharge, by country (19,350). **Supplementary Table 2.** Intrapersonal factors of the mothers who reported partial or no breastfeeding at the time of discharge, by country (7,359).
