# Supplementary material for: Factors associated with exclusive breastfeeding at discharge during the COVID-19 pandemic in 17 WHO European Region countries
Source: Int Breastfeed J. 2022 Dec 2;17:83. doi: 10.1186/s13006-022-00517-1 (PMC9716162; doi:10.1186/s13006-022-00517-1)
Supplement: Supplementary file 1 — Additional file 1: Supplementary Table 1. Intrapersonal factors of the mothers who reported exclusive breastfeeding at the time of discharge, by country (19,350). Supplementary Table 2. Intrapersonal factors of the mothers who reported partial or no breastfeeding at the time of discharge, by country (7,359). [file 13006_2022_517_MOESM1_ESM.docx]

Factors associated with exclusive breastfeeding at discharge during the COVID-19 pandemic in 17 WHO European Region countries

SUPPLEMENTARY FILE

**Table of Contents**

[Supplementary Table 1. Intrapersonal factors of the mothers who reported exclusive breastfeeding at the time of discharge, by country (19,350). 2](#_Toc117250067)

[Supplementary Table 2. Intrapersonal factors of the mothers who reported partial or no breastfeeding at the time of discharge, by country (7,359). 4](#_Toc117250068)

# Supplementary Table 1. Intrapersonal factors of the mothers who reported exclusive breastfeeding at the time of discharge by country (19,350).

|  | Bosnia and Herzegovina | Croatia | France | Germany | Italy | Latvia | Lithuania | Luxemburg | Norway | Poland | Portugal | Romania | Serbia | Slovenia | Spain | Sweden | Switzerland |
| --- | --- | --- | --- | --- | --- | --- | --- | --- | --- | --- | --- | --- | --- | --- | --- | --- | --- |
| N | 227 | 836 | 649 | 687 | 4109 | 1186 | 682 | 292 | 2178 | 1152 | 1372 | 413 | 466 | 1212 | 231 | 2966 | 692 |
|  | n (%) | n (%) | n (%) | n (%) | n (%) | n (%) | n (%) | n (%) | n (%) | n (%) | n (%) | n (%) | n (%) | n (%) | n (%) | n (%) | n (%) |
| ***Sociodemographic factors*** | | | | | | | | | | | | | | | | | |
| *Maternal age* |  |  |  |  |  |  |  |  |  |  |  |  |  |  |  |  |  |
| 18-24 | 37 (16.3) | 42 (5.0) | 15 (2.3) | 20 (2.9) | 84 (2.0) | 119 (10.0) | 69 (10.1) | 4 (1.4) | 125 (5.7) | 30 (2.6) | 49 (3.6) | 27 (6.5) | 27 (5.8) | 108 (8.9) | 0 (0.0) | 144 (4.9) | 10 (1.4) |
| 25-30 | 99 (43.6) | 294 (35.2) | 207 (31.9) | 184 (26.8) | 908 (22.1) | 536 (45.2) | 345 (50.6) | 72 (24.7) | 960 (44.1) | 461 (40.0) | 427 (31.1) | 171 (41.4) | 157 (33.7) | 553 (45.6) | 39 (16.9) | 1274 (43.0) | 152 (22.0) |
| 31-35 | 72 (31.7) | 341 (40.8) | 295 (45.5) | 319 (46.4) | 1795 (43.7) | 389 (32.8) | 224 (32.8) | 147 (50.3) | 864 (39.7) | 493 (42.8) | 581 (42.3) | 168 (40.7) | 172 (36.9) | 422 (34.8) | 96 (41.6) | 1192 (40.2) | 344 (49.7) |
| 36-39 | 15 (6.6) | 128 (15.3) | 109 (16.8) | 126 (18.3) | 974 (23.7) | 122 (10.3) | 39 (5.7) | 61 (20.9) | 189 (8.7) | 139 (12.1) | 255 (18.6) | 38 (9.2) | 82 (17.6) | 109 (9.0) | 70 (30.3) | 301 (10.1) | 144 (20.8) |
| 40 or older | 4 (1.8) | 31 (3.7) | 23 (3.5) | 38 (5.5) | 348 (8.5) | 20 (1.7) | 5 (0.7) | 8 (2.7) | 40 (1.8) | 29 (2.5) | 60 (4.4) | 9 (2.2) | 28 (6.0) | 20 (1.7) | 26 (11.3) | 55 (1.9) | 42 (6.1) |
| *Parity* |  |  |  |  |  |  |  |  |  |  |  |  |  |  |  |  |  |
| Primipara | 87 (38.3) | 398 (47.6) | 304 (46.8) | 292 (42.5) | 2653 (64.6) | 577 (48.7) | 445 (65.2) | 150 (51.4) | 1183 (54.3) | 536 (46.5) | 869 (63.3) | 202 (48.9) | 199 (42.7) | 602 (49.7) | 145 (62.8) | 1645 (55.5) | 288 (41.6) |
| Multipara | 140 (61.7) | 438 (52.4) | 345 (53.2) | 395 (57.5) | 1456 (35.4) | 609 (51.3) | 237 (34.8) | 142 (48.6) | 995 (45.7) | 616 (53.5) | 503 (36.7) | 211 (51.1) | 267 (57.3) | 610 (50.3) | 86 (37.2) | 1321 (44.5) | 404 (58.4) |
| *Maternal education* | | |  |  |  |  |  |  |  |  |  |  |  |  |  |  |  |
| Junior high school or lower | 22 (9.7) | 56 (6.7) | 1 (0.2) | 87 (12.7) | 172 (4.2) | 128 (10.8) | 48 (7.0) | 12 (4.1) | 30 (1.4) | 5 (0.4) | 2 (0.1) | 5 (1.2) | 5 (1.1) | 293 (24.2) | 3 (1.3) | 32 (1.1) | 44 (6.4) |
| High school | 76 (33.5) | 182 (21.8) | 96 (14.8) | 162 (23.6) | 1547 (37.6) | 156 (13.2) | 56 (8.2) | 50 (17.1) | 421 (19.3) | 101 (8.8) | 306 (22.3) | 63 (15.3) | 108 (23.2) | 90 (7.4) | 16 (6.9) | 735 (24.8) | 148 (21.4) |
| University degree | 94 (41.4) | 429 (51.3) | 183 (28.2) | 157 (22.9) | 1234 (30.0) | 533 (44.9) | 152 (22.3) | 101 (34.6) | 1106 (50.8) | 23 (2.0) | 483 (35.2) | 167 (40.4) | 198 (42.5) | 587 (48.4) | 102 (44.2) | 1725 (58.2) | 253 (36.6) |
| Graduate degree (Master/Doctorate) | 35 (15.4) | 169 (20.2) | 369 (56.9) | 281 (40.9) | 1156 (28.1) | 369 (31.1) | 426 (62.5) | 129 (44.2) | 621 (28.5) | 1023 (88.8) | 581 (42.3) | 178 (43.1) | 155 (33.3) | 242 (20.0) | 110 (47.6) | 474 (16.0) | 247 (35.7) |
| *Payment of maternity care (insurance status)* | | | | | | |  |  |  |  |  |  |  |  |  |  |  |
| Non-private insurance (public/state/employer) | 224 (98.7) | 831 (99.4) | 545 (84.0) | 678 (98.7) | 4042 (98.4) | 1168 (98.5) | 680 (99.7) | 284 (97.3) | 2178 (100.0) | 1135 (98.5) | 1151 (83.9) | 323 (78.2) | 452 (97.0) | 1212 (100.0) | 183 (79.2) | 2963 (99.9) | 585 (84.5) |
| Private insurance | 0 (0.0) | 0 (0.0) | 84 (12.9) | 7 (1.0) | 18 (0.4) | 3 (0.3) | 0 (0.0) | 8 (2.7) | 0 (0.0) | 2 (0.2) | 176 (12.8) | 12 (2.9) | 5 (1.1) | 0 (0.0) | 48 (20.8) | 0 (0.0) | 104 (15.0) |
| No insurance (self-paid) | 3 (1.3) | 5 (0.6) | 20 (3.1) | 2 (0.3) | 49 (1.2) | 15 (1.3) | 2 (0.3) | 0 (0.0) | 0 (0.0) | 15 (1.3) | 45 (3.3) | 78 (18.9) | 9 (1.9) | 0 (0.0) | 0 (0.0) | 3 (0.1) | 3 (0.4) |
| *Infant born in same country as mother’s origin* | | | | | | |  |  |  |  |  |  |  |  |  |  |  |
| Yes | 210 (92.5) | 760 (90.9) | 599 (92.3) | 582 (84.7) | 3929 (95.6) | 1168 (98.5) | 666 (97.7) | 176 (60.3) | 2002 (91.9) | 1147 (99.6) | 1279 (93.2) | 404 (97.8) | 425 (91.2) | 1165 (96.1) | 209 (90.5) | 2767 (93.3) | 501 (72.4) |
| No | 17 (7.5) | 76 (9.1) | 50 (7.7) | 105 (15.3) | 180 (4.4) | 18 (1.5) | 16 (2.3) | 116 (39.7) | 176 (8.1) | 5 (0.4) | 93 (6.8) | 9 (2.2) | 41 (8.8) | 47 (3.9) | 22 (9.5) | 199 (6.7) | 191 (27.6) |
| ***Prenatal and birth factors*** | | | | |  |  |  |  |  |  |  |  |  |  |  |  |  |
| *Mode of birth* | | | |  |  |  |  |  |  |  |  |  |  |  |  |  |  |
| Spontaneous vaginal birth | 179 (78.9) | 706 (84.4) | 485 (74.7) | 512 (74.5) | 3050 (74.2) | 961 (81.0) | 568 (83.3) | 211 (72.3) | 1758 (80.7) | 750 (65.1) | 738 (53.8) | 217 (52.5) | 341 (73.2) | 1053 (86.9) | 163 (70.6) | 2575 (86.8) | 495 (71.5) |
| Instrumental vaginal birth | 3 (1.3) | 18 (2.2) | 85 (13.1) | 56 (8.2) | 290 (7.1) | 68 (5.7) | 19 (2.8) | 39 (13.4) | 228 (10.5) | 21 (1.8) | 347 (25.3) | 9 (2.2) | 10 (2.1) | 39 (3.2) | 29 (12.6) | 189 (6.4) | 72 (10.4) |
| Cesarean birth | 45 (19.8) | 112 (13.4) | 79 (12.2) | 119 (17.3) | 769 (18.7) | 157 (13.2) | 95 (13.9) | 42 (14.4) | 192 (8.8) | 381 (33.1) | 287 (20.9) | 187 (45.3) | 115 (24.7) | 120 (9.9) | 39 (16.9) | 202 (6.8) | 125 (18.1) |
| *Difficulty attending prenatal care* | | | | | |  |  |  |  |  |  |  |  |  |  |  |  |
| Yes, always/Nearly always | 50 (22.0) | 44 (5.3) | 33 (5.1) | 24 (3.5) | 270 (6.6) | 38 (3.2) | 81 (11.9) | 21 (7.2) | 147 (6.7) | 74 (6.4) | 146 (10.6) | 63 (15.3) | 94 (20.2) | 110 (9.1) | 35 (15.2) | 132 (4.5) | 18 (2.6) |
| Sometimes | 105 (46.3) | 218 (26.1) | 158 (24.3) | 155 (22.6) | 1401 (34.1) | 223 (18.8) | 217 (31.8) | 74 (25.3) | 709 (32.6) | 330 (28.6) | 452 (32.9) | 165 (40.0) | 183 (39.3) | 420 (34.7) | 105 (45.5) | 739 (24.9) | 111 (16.0) |
| No, never/Almost never | 72 (31.7) | 574 (68.7) | 458 (70.6) | 508 (73.9) | 2438 (59.3) | 925 (78.0) | 384 (56.3) | 197 (67.5) | 1322 (60.7) | 748 (64.9) | 774 (56.4) | 185 (44.8) | 189 (40.6) | 682 (56.3) | 91 (39.4) | 2095 (70.6) | 563 (81.4) |
| *Faced barriers to access prenatal care* | | | | |  |  |  |  |  |  |  |  |  |  |  |  |  |
| Yes, always/Nearly always | 33 (14.5) | 33 (3.9) | 16 (2.5) | 44 (6.4) | 216 (5.3) | 33 (2.8) | 54 (7.9) | 18 (6.2) | 103 (4.7) | 61 (5.3) | 85 (6.2) | 33 (8.0) | 67 (14.4) | 60 (5.0) | 13 (5.6) | 126 (4.2) | 15 (2.2) |
| Sometimes | 105 (46.3) | 211 (25.2) | 95 (14.6) | 169 (24.6) | 958 (23.3) | 232 (19.6) | 178 (26.1) | 61 (20.9) | 540 (24.8) | 257 (22.3) | 337 (24.6) | 135 (32.7) | 175 (37.6) | 278 (22.9) | 54 (23.4) | 709 (23.9) | 92 (13.3) |
| No, never/Almost never | 89 (39.2) | 592 (70.8) | 538 (82.9) | 474 (69.0) | 2935 (71.4) | 921 (77.7) | 450 (66.0) | 213 (72.9) | 1535 (70.5) | 834 (72.4) | 950 (69.2) | 245 (59.3) | 224 (48.1) | 874 (72.1) | 164 (71.0) | 2131 (71.8) | 585 (84.5) |
| *Maternal ICU admission* | | |  |  |  |  |  |  |  |  |  |  |  |  |  |  |  |
| Yes | 3 (1.3) | 4 (0.5) | 0 (0.0) | 0 (0.0) | 3 (0.1) | 0 (0.0) | 6 (0.9) | 0 (0.0) | 6 (0.3) | 0 (0.0) | 1 (0.1) | 7 (1.7) | 4 (0.9) | 51 (4.2) | 1 (0.4) | 5 (0.2) | 1 (0.1) |

Abbreviations: BFHI = Baby Friendly Hospital Initiative; HCP = health care provider.

Note: exclusive breastfeeding was assessed from maternal report of exclusive breastfeeding at the time of discharge (with no provision of formula).

# Supplementary Table 2. Intrapersonal factors of the mothers who reported partial or no breastfeeding at the time of discharge by country (7,359).

|  | Bosnia and Herzegovina | Croatia | France | Germany | Italy | Latvia | Lithuania | Luxemburg | Norway | Poland | Portugal | Romania | Serbia | Slovenia | Spain | Sweden | Switzerland |
| --- | --- | --- | --- | --- | --- | --- | --- | --- | --- | --- | --- | --- | --- | --- | --- | --- | --- |
| N | 227 | 836 | 649 | 687 | 4109 | 1186 | 682 | 292 | 2178 | 1152 | 1372 | 413 | 466 | 1212 | 231 | 2966 | 692 |
|  | n (%) | n (%) | n (%) | n (%) | n (%) | n (%) | n (%) | n (%) | n (%) | n (%) | n (%) | n (%) | n (%) | n (%) | n (%) | n (%) | n (%) |
| ***Sociodemographic factors*** | | | | | | | | | | | | | | | | | |
| *Maternal age* |  |  |  |  |  |  |  |  |  |  |  |  |  |  |  |  |  |
| 18-24 | 29 (22.0) | 50 (7.0) | 31 (7.8) | 12 (5.6) | 29 (1.7) | 50 (11.3) | 27 (16.2) | 2 (1.9) | 40 (7.9) | 17 (6.1) | 15 (3.9) | 27 (6.2) | 21 (7.7) | 53 (8.8) | 1 (1.9) | 49 (6.0) | 3 (1.9) |
| 25-30 | 54 (40.9) | 283 (39.4) | 150 (37.8) | 58 (26.9) | 352 (21.2) | 191 (43.2) | 89 (53.3) | 33 (31.1) | 215 (42.2) | 112 (40.0) | 115 (29.7) | 169 (38.8) | 81 (29.8) | 276 (45.7) | 10 (19.2) | 367 (44.6) | 31 (20.0) |
| 31-35 | 38 (28.8) | 282 (39.2) | 155 (39.0) | 91 (42.1) | 679 (40.9) | 139 (31.4) | 45 (26.9) | 41 (38.7) | 186 (36.5) | 116 (41.4) | 170 (43.9) | 172 (39.4) | 105 (38.6) | 190 (31.5) | 19 (36.5) | 298 (36.2) | 81 (52.3) |
| 36-39 | 9 (6.8) | 75 (10.4) | 50 (12.6) | 43 (19.9) | 433 (26.1) | 53 (12.0) | 6 (3.6) | 24 (22.6) | 58 (11.4) | 27 (9.6) | 61 (15.8) | 55 (12.6) | 41 (15.1) | 67 (11.1) | 13 (25.0) | 87 (10.6) | 28 (18.1) |
| 40 or older | 2 (1.5) | 29 (4.0) | 11 (2.8) | 12 (5.6) | 169 (10.2) | 9 (2.0) | 0 (0.0) | 6 (5.7) | 10 (2.0) | 8 (2.9) | 26 (6.7) | 13 (3.0) | 24 (8.8) | 18 (3.0) | 9 (17.3) | 22 (2.7) | 12 (7.7) |
| *Parity* |  |  |  |  |  |  |  |  |  |  |  |  |  |  |  |  |  |
| Primipara | 94 (71.2) | 483 (67.2) | 225 (56.7) | 135 (62.5) | 1273 (76.6) | 278 (62.9) | 131 (78.4) | 71 (67.0) | 370 (72.7) | 214 (76.4) | 310 (80.1) | 342 (78.4) | 157 (57.7) | 404 (66.9) | 43 (82.7) | 559 (67.9) | 100 (64.5) |
| Multipara | 38 (28.8) | 236 (32.8) | 172 (43.3) | 81 (37.5) | 389 (23.4) | 164 (37.1) | 36 (21.6) | 35 (33.0) | 139 (27.3) | 66 (23.6) | 77 (19.9) | 94 (21.6) | 115 (42.3) | 200 (33.1) | 9 (17.3) | 264 (32.1) | 55 (35.5) |
| *Maternal education* | | |  |  |  |  |  |  |  |  |  |  |  |  |  |  |  |
| Junior high school or lower | 7 (5.3) | 41 (5.7) | 4 (1.0) | 58 (26.9) | 82 (4.9) | 50 (11.3) | 21 (12.6) | 6 (5.7) | 14 (2.8) | 0 (0.0) | 0 (0.0) | 1 (0.2) | 2 (0.7) | 193 (32.0) | 5 (9.6) | 16 (1.9) | 18 (11.6) |
| High school | 41 (31.1) | 186 (25.9) | 115 (29.0) | 43 (19.9) | 691 (41.6) | 66 (14.9) | 19 (11.4) | 33 (31.1) | 117 (23.0) | 38 (13.6) | 91 (23.5) | 44 (10.1) | 80 (29.4) | 40 (6.6) | 7 (13.5) | 264 (32.1) | 41 (26.5) |
| University degree | 59 (44.7) | 345 (48.0) | 136 (34.3) | 38 (17.6) | 444 (26.7) | 185 (41.9) | 35 (21.0) | 32 (30.2) | 265 (52.1) | 4 (1.4) | 124 (32.0) | 173 (39.7) | 126 (46.3) | 287 (47.5) | 20 (38.5) | 421 (51.2) | 41 (26.5) |
| Graduate degree (Master/Doctorate) | 25 (18.9) | 147 (20.4) | 142 (35.8) | 77 (35.6) | 445 (26.8) | 141 (31.9) | 92 (55.1) | 35 (33.0) | 113 (22.2) | 238 (85.0) | 172 (44.4) | 218 (50.0) | 64 (23.5) | 84 (13.9) | 20 (38.5) | 122 (14.8) | 55 (35.5) |
| *Payment of maternity care (insurance status)* | | | | | | |  |  |  |  |  |  |  |  |  |  |  |
| Non-private insurance (public/state/employer) | 129 (97.7) | 714 (99.3) | 308 (77.6) | 215 (99.5) | 1608 (96.8) | 435 (98.4) | 165 (98.8) | 104 (98.1) | 509 (100.0) | 276 (98.6) | 286 (73.9) | 309 (70.9) | 260 (95.6) | 604 (100.0) | 39 (75.0) | 822 (99.9) | 130 (83.9) |
| Private insurance | 0 (0.0) | 1 (0.1) | 72 (18.1) | 1 (0.5) | 9 (0.5) | 0 (0.0) | 0 (0.0) | 2 (1.9) | 0 (0.0) | 0 (0.0) | 77 (19.9) | 7 (1.6) | 1 (0.4) | 0 (0.0) | 13 (25.0) | 0 (0.0) | 25 (16.1) |
| No insurance (self-paid) | 3 (2.3) | 4 (0.6) | 17 (4.3) | 0 (0.0) | 45 (2.7) | 7 (1.6) | 2 (1.2) | 0 (0.0) | 0 (0.0) | 4 (1.4) | 24 (6.2) | 120 (27.5) | 11 (4.0) | 0 (0.0) | 0 (0.0) | 1 (0.1) | 0 (0.0) |
| *Infant born in same country as mother’s origin* | | | | | | |  |  |  |  |  |  |  |  |  |  |  |
| Yes | 122 (92.4) | 657 (91.4) | 380 (95.7) | 176 (81.5) | 1584 (95.3) | 438 (99.1) | 161 (96.4) | 71 (67.0) | 464 (91.2) | 278 (99.3) | 352 (91.0) | 427 (97.9) | 252 (92.6) | 574 (95.0) | 48 (92.3) | 745 (90.5) | 108 (69.7) |
| No | 10 (7.6) | 62 (8.6) | 17 (4.3) | 40 (18.5) | 78 (4.7) | 4 (0.9) | 6 (3.6) | 35 (33.0) | 45 (8.8) | 2 (0.7) | 35 (9.0) | 9 (2.1) | 20 (7.4) | 30 (5.0) | 4 (7.7) | 78 (9.5) | 47 (30.3) |
| ***Prenatal and birth factors*** | | | | |  |  |  |  |  |  |  |  |  |  |  |  |  |
| *Mode of birth* | | | |  |  |  |  |  |  |  |  |  |  |  |  |  |  |
| Spontaneous vaginal birth | 84 (63.6) | 532 (74.0) | 270 (68.0) | 129 (59.7) | 964 (58.0) | 308 (69.7) | 126 (75.4) | 61 (57.5) | 334 (65.6) | 148 (52.9) | 120 (31.0) | 129 (29.6) | 174 (64.0) | 448 (74.2) | 23 (44.2) | 604 (73.4) | 93 (60.0) |
| Instrumental vaginal birth | 0 (0.0) | 21 (2.9) | 66 (16.6) | 22 (10.2) | 104 (6.3) | 31 (7.0) | 6 (3.6) | 14 (13.2) | 70 (13.8) | 10 (3.6) | 101 (26.1) | 1 (0.2) | 7 (2.6) | 25 (4.1) | 13 (25.0) | 81 (9.8) | 20 (12.9) |
| Cesarean birth | 48 (36.4) | 166 (23.1) | 61 (15.4) | 65 (30.1) | 594 (35.7) | 103 (23.3) | 35 (21.0) | 31 (29.2) | 105 (20.6) | 122 (43.6) | 166 (42.9) | 306 (70.2) | 91 (33.5) | 131 (21.7) | 16 (30.8) | 138 (16.8) | 42 (27.1) |
| *Difficulty attending prenatal care* | | | | | |  |  |  |  |  |  |  |  |  |  |  |  |
| Yes, always/Nearly always | 35 (26.5) | 55 (7.6) | 21 (5.3) | 15 (6.9) | 114 (6.9) | 12 (2.7) | 19 (11.4) | 9 (8.5) | 43 (8.4) | 17 (6.1) | 37 (9.6) | 31 (7.1) | 46 (16.9) | 78 (12.9) | 4 (7.7) | 46 (5.6) | 3 (1.9) |
| Sometimes | 58 (43.9) | 214 (29.8) | 88 (22.2) | 39 (18.1) | 560 (33.7) | 90 (20.4) | 56 (33.5) | 32 (30.2) | 192 (37.7) | 89 (31.8) | 137 (35.4) | 154 (35.3) | 121 (44.5) | 202 (33.4) | 24 (46.2) | 238 (28.9) | 28 (18.1) |
| No, never/Almost never | 39 (29.5) | 450 (62.6) | 288 (72.5) | 162 (75.0) | 988 (59.4) | 340 (76.9) | 92 (55.1) | 65 (61.3) | 274 (53.8) | 174 (62.1) | 213 (55.0) | 251 (57.6) | 105 (38.6) | 324 (53.6) | 24 (46.2) | 539 (65.5) | 124 (80.0) |
| *Faced barriers to access prenatal care* | | | | |  |  |  |  |  |  |  |  |  |  |  |  |  |
| Yes, always/Nearly always | 22 (16.7) | 31 (4.3) | 12 (3.0) | 12 (5.6) | 90 (5.4) | 12 (2.7) | 16 (9.6) | 10 (9.4) | 39 (7.7) | 6 (2.1) | 27 (7.0) | 28 (6.4) | 36 (13.2) | 39 (6.5) | 4 (7.7) | 50 (6.1) | 4 (2.6) |
| Sometimes | 54 (40.9) | 217 (30.2) | 49 (12.3) | 45 (20.8) | 374 (22.5) | 105 (23.8) | 40 (24.0) | 19 (17.9) | 137 (26.9) | 72 (25.7) | 103 (26.6) | 107 (24.5) | 100 (36.8) | 156 (25.8) | 7 (13.5) | 176 (21.4) | 16 (10.3) |
| No, never/Almost never | 56 (42.4) | 471 (65.5) | 336 (84.6) | 159 (73.6) | 1198 (72.1) | 325 (73.5) | 111 (66.5) | 77 (72.6) | 333 (65.4) | 202 (72.1) | 257 (66.4) | 301 (69.0) | 136 (50.0) | 409 (67.7) | 41 (78.8) | 597 (72.5) | 135 (87.1) |
| *Maternal ICU admission* | | |  |  |  |  |  |  |  |  |  |  |  |  |  |  |  |
| Yes | 0 (0.0) | 4 (0.6) | 0 (0.0) | 1 (0.5) | 3 (0.2) | 1 (0.2) | 1 (0.6) | 0 (0.0) | 11 (2.2) | 0 (0.0) | 1 (0.3) | 21 (4.8) | 3 (1.1) | 37 (6.1) | 0 (0.0) | 1 (0.1) | 0 (0.0) |

Abbreviations: BFHI = Baby Friendly Hospital Initiative; HCP = health care provider.

Note: partial or no breastfeeding was assessed from maternal report of breastfeeding at the time of discharge (with provision of formula).
